# Plasticity of Escape Responses: Prior Predator Experience Enhances Escape Performance in a Coral Reef Fish

**DOI:** 10.1371/journal.pone.0132790

**Published:** 2015-08-05

**Authors:** Ryan A. Ramasamy, Bridie J. M. Allan, Mark I. McCormick

**Affiliations:** ARC Centre of Excellence for Coral Reef Studies and College of Marine and Environmental Sciences, James Cook University, Townsville, Queensland, Australia; University of Windsor, CANADA

## Abstract

Teleost and amphibian prey undertake fast-start escape responses during a predatory attack in an attempt to avoid being captured. Although previously viewed as a reflex reaction controlled by the autonomic nervous system, the escape responses of individuals when repeatedly startled are highly variable in their characteristics, suggesting some behavioural mediation of the response. Previous studies have shown that fishes are able to learn from past experiences, but few studies have assessed how past experience with predators affect the fast-start response. Here we determined whether prior experience with the smell or sight of a predator (the Dottyback, *Pseudochromis fuscus*) affected the escape response of juveniles of the Spiny Chromis (*Acanthochromis polyacanthus*). Results show that individuals exposed to any of the predator cues prior to being startled exhibited a stronger escape response (i.e., reduced latency, increased escape distance, mean response speed, maximum response speed and maximum acceleration) when compared with controls. This study demonstrates the plasticity of escape responses and highlights the potential for naïve reef fish to take into account both visual and olfactory threat cues simultaneously to optimise the amplitude of their kinematic responses to perceived risk.

## Introduction

Most organisms live under a constant threat of predation, and this threat is highest during the early life stages when the capacity for prey to detect and avoid or evade predation is low [1]. There are strong selective forces for prey to efficiently detect and escape predators, but escape can be energetically costly so there may be some pressure for prey to modulate their escape response to match the level of threat posed by the predator. Small changes to the effectiveness of any step in the escape response can lead to large changes in the overall probability of successful escape for prey individuals [2–3].

Fish detect and assess risk through their sensory systems, including visual [4], auditory [5], mechanoreception and olfactory senses [6]. In the event of an attack, prey exhibit an escape response that usually involves a fast-start response. Fast-starts are short high-energy swimming bursts that are powered anaerobically [2]. They are elicited by the Mauthner neurons and are used to evade predation. Fast-starts were once commonly referred to as a stereotypic (i.e. consistent), hard-wired response [7]. This view has changed over the past decade with authors showing variability in the escape response within species [8–12]. Interestingly, the few studies that have examined within-individual variation in fast-start responses have found considerable levels of variation (exceeding 40%) in many of the kinematic aspects that characterise the consecutive bursts [13–14]. This individual variability in escape responses is ecologically meaningful, as responding with maximum effort regardless of the relative risk is an energetically costly strategy due to the anaerobically costly process of high speed swimming and disruption to other fitness-related behaviours such as foraging.

The “Economic Hypothesis” described by Ydenberg and Dill [15] suggests that prey may alter their behaviour to minimise costly activities such as escape responses. The high cost of repaying oxygen debts from anaerobic metabolism [16], and the metabolic (e.g., stress) and behavioural disruption associated with escape responses, suggests that it could be beneficial for animals to alter the intensity of their fast-start to match the intensity of the threat and in doing so minimise its metabolic cost. Moreover, research on risk assessment suggests that prey should attempt to optimise their response to risk by using information from all possible sensory systems relevant to the potential threat [17]. Aquatic organisms respond conservatively to olfactory information on threats (e.g., damage-released alarm cues that are released upon mechanical damage to the epidermis [18]), which can be fine-tuned using information from other senses such as vision [19] and mechanoreception [20]. Thus we predict that a prey’s fast-start response should be responsive to the level of the perceived threat, which will be directly influenced by the amount of information available on which to judge risk. Therefore, our study aimed to test whether information concerning the proximity of a predation threat affected the fast-start responses of a juvenile fish, the Spiny Chromis, *Acanthochromis polyacanthus* (Pomacentridae). Specifically, we investigated two questions: (1) Are *A*. *polyacanthus* individuals consistent in their fast-start responses? (2) Does the perceived risk level influence the strength of the preys fast-start response?

## Materials and Methods

### Ethics statement

Research was carried out under approval of the James Cook University animal ethics committee (permit: A2005) and according to the University's animal ethics guidelines. Fish collections around Lizard Island, Great Barrier Reef were carried out with permission of the Great Barrier Reef Marine Park’s Authority (permit: G12/34811.1).

### Study species

The prey species used was the Spiny Chromis, *Acanthochromis polyacanthus* (Pomacentridae), a reef-associated brooding planktivore commonly found on the Great Barrier Reef (GBR), Australia. To ensure genetic diversity, 90 juveniles (mean SL ± se: 19.03 mm ± 0.13 mm; ~ 45d post-hatching) from 5 separate schools were collected on SCUBA with hand nets from Lizard Island in the northern Great Barrier Reef (GBR), Australia (14°40′08″S 145°27′34″E). Experiments were conducted during November 2014 at the Lizard Island Research Station. Juveniles were randomly allocated to groups of 30, maintained in 32 L tanks (432 x 324 x 305 mm) at ambient conditions (28.2°C ± 0.2°C) and fed *ab libitum* with *Artemia naupili*. Prey were starved for 16 h prior to trials commencing to control for satiation.

The predator used was the dottyback, *Pseudochromis fuscus* (Pseudochromidae) (mean SL ± se: 74.21 mm ± 0.93), which was collected on SCUBA using clove oil and held in a 20 L tank (432 x 324 x 203 mm). This species is a common mesopredator found throughout the GBR and is an important predator of newly settled reef fishes [21]. To avoid the presence of diet cues from faeces being present in the odour, *P*. *fuscus* was fed pieces of squid on the days predator odour was not required.

### Conditioning treatment

Prior to trials commencing, individuals were taught to recognise the predator as a threat by exposing *A*. *polyacanthus* juveniles to the sight and odour of the predator at the same time as damage-released chemical alarm cues from a conspecific. Chemical alarm cues were obtained through 5 superficial cuts to the epidermis on both sides of 4 *A*. *polyacanthus* individuals, culled by cold shock, and rinsed with 15 ml of seawater (following the protocol of Lönnstedt and McCormick [22]). This predator-training process was undertaken for each of the 3 holding tanks. This coupling of the visual and/or odour cues with a chemical alarm cue leads to the assignment of risk to the cues through a process known as associative learning [17]. To couple odour with the visual cue, a *P*. *fuscus* was placed into a transparent 5 L plastic tank, which was then placed into the *A*. *polyacanthus* holding tank. In this way the *P*. *fuscus* could be readily seen by the resident *A*. *polyacanthus* juveniles, but the *P*. *fuscus* could not escape to eat them. Simultaneously, 30 ml *P*. *fuscus* odour (prepared by turning off the inflow of water for 16 h with *P*. *fuscus* freely swimming in a 32 L tank with an airstone) and 15 ml of chemical alarm cues from *A*. *polyacanthus* were injected through plastic tubing into the holding tank. Juvenile *A*. *polyacanthus* were exposed to the cues for 30 min (as per the protocol of Lönnstedt et al. [23]).

### Fast-start arena and protocol

The testing arena consisted of a transparent circular acrylic arena (diameter 200mm; height 70mm) contained within a large opaque-sided plastic tank (585 x 420 x 330mm; 60 L) with a transparent Perspex bottom to allow responses to be filmed from below. To avoid aggressive interactions between the predator and the prey, the prey fish was contained within the circular acrylic arena. This allowed the prey to receive visual cues from the presence of the predator. To minimise vertical displacement of the prey during the escape response, the water level was set at 70mm.

A single prey fish was placed into the circular arena then one of four treatments were applied after a 5-minute acclimation period: a) Predator odour (n = 15), b) predator visual (n = 15), c) combination of predator visual and odour (n = 15), d) control (n = 15). Predator odour consisted of 15 ml of water from the predator tank slowly injected into the inner arena following the introduction of an empty 5 L plastic tank (210 x 120 x 115 mm) filled with seawater. To achieve the visual cue, a predator was placed into a 5 L plastic tank and slowly introduced into the outer arena before the 15 ml of seawater was injected. The control consisted of 15 ml of seawater injected into the inner arena following the introduction of an empty 5 L plastic tank filled with seawater.

Fast-start responses were elicited after a 5-minute exposure period to the treatment cue by the release of a weighted test tube with a tapered end into the testing arena. The weighted test tube was controlled by a piece of fishing line that was long enough such that the tapered tip of the test tube only just touched the surface of the water. To avoid a premature escape response associated with visual stimulation occurring, the test tube was released from above into a 550 mm piece of 40 mm diameter PVC pipe. To standardise for fish position relative to the stimulus, fast starts were only elicited when the fish was close to the PVC pipe. To test for consistency in fast-start performance within individuals, fish were startled 3 times. To reduce the build-up of lactic acid across fast-starts for individuals, which could affect performance [24], fish were given 3 min intervals between startles. To avoid a build-up of chemical cues, the arena was emptied and rinsed before the next trial commenced.

Prey escape variables were only measured when prey performed a C-start (commencement of fast-start that results in the individual forming a C-shape). Escape responses were recorded at 480 frames per second (Casio EX-ZR1000) as a silhouette from below obtained through pointing the camera at a mirror angled at 45° below the arena. To minimise visual disturbances, black sheeting surrounded the front of the mirror so that any movement within the room was undetected by the fish. A 1cm line was drawn in the centre of the inner arena to enable calibration for video analysis.

### Kinematic variables

Kinematic variables associated with the fast-start response were analysed using the image-analysis software Image-J, with a manual tracking plug-in. The point where each fish was tracked was standardised by following the same point on each fish (i.e. the position directly behind the eyes which corresponds to the thickest part of the body). We choose to standardise tracking based on this point of the body as it the most stable and easiest to track owing to the small size of the larvae. The following kinematic variables were measured:

1. Response latency (s) was measured as the time interval between the stimulus touching the water surface and the first detectable movement of the fish.
2. Response duration (s) was measured as the elapsed time from the start to the end of the escape response (i.e., when the prey comes to a halt).
3. Response distance (m) is a measure of the total distance covered by the fish from the onset of the response to when the response ends (i.e., when the prey comes to a halt).
4. Mean response speed (m s^-1^) was measured as the distance covered within a fixed time (first 24 ms after initial response) which corresponds to the average duration of the first two tail flips (the first two axial bends, i.e. stages 1 and 2 based on Domenici & Blake [2]). This period is considered crucial for avoiding predator ambush attacks [2, 21].
5. Maximum response speed (m s^-1^) was measured as the maximum speed reached at any time during the response.
6. Maximum acceleration (m s^-2^) was measured as the maximum acceleration within a fixed time (first 24 ms after initial response).

### Statistical analyses

To ensure that there was no difference between trials in the distance between fish and the stimulus tube, a one-factor analysis of variance was carried out in STATISTICA (v12). To quantify the levels of variability for each kinematic variable among consecutive fast-starts coefficients of variation were calculated for each individual from the control treatments. To determine whether there was a change in kinematics among three consecutive fast-starts a paired sample t-test was undertaken on the values from the first and third burst in STATISTICA (v12). Variables (response distance, mean response speed and maximum acceleration) were log10(x) transformed to improve normality and homogeneity of variance.

A one-factor multivariate analysis of variance (MANOVA) that included the strongest response in all 6 kinematic variables was undertaken to test whether there was a difference in the kinematic response of fish among treatments (n = 60). This was followed by one-factor analysis of variances (ANOVAs) and Tukey’s HSD post-hoc tests to determine the nature of the significant difference found by MANOVA. Data were examined for the assumptions of homogeneity of variance and normality using residual analysis. The effect sizes (*d*) of the overall ANOVAs were expressed as Cohen’s *d* statistics.

## Results

No statistical difference was found in the distance between fish and the stimulus tube among treatments (F_3, 153_ = 1.67, p = 0.176). There were considerable levels of variability among consecutive bursts within individuals for all kinematic variables measured (Table 1). Latency to respond was the most variable with a within-individual CVs of 4.9 to 140% (mean 36.7%). No trend for an increase or decrease in kinematics between the first and last fast-start was evident (Latency: t_97_ = -0.74, p = 0.464; Response duration: t_97_ = 0.16, p = 0.156; Response distance: t_97_ = 0.41, p = 0.683; Mean response speed: t_97_ = 1.1, p = 0.27; Maximum response speed: t_97_ = -1.47, p = 0.145; Maximum acceleration: t_97_ = -1.37, p = 0.172).

**Table 1:** Kinematics of the escape response of *Acanthochromis polyacanthus* juveniles indicating their mean response, mean coefficient of variation (CV) and range of CV at the individual level estimated from 3 consecutive bursts per fish.

Variable	Mean	Mean CV	Range of CV for individuals
**Latency**	0.015 s	36.7%	4.9–140.0%
**Response duration**	0.172 s	37.0%	6.9–76.0%
**Response distance**	0.073 m	37.1%	0.9–67.6%
**Mean response speed**	1.197 m/s	31.3%	5.3–84.8%
**Maximum response speed**	1.676 m/s/s	32.1%	2.8–40.5%
**Maximum acceleration**	0.901 m/s	23.4%	1.1–105.4%

**Fig 1:**
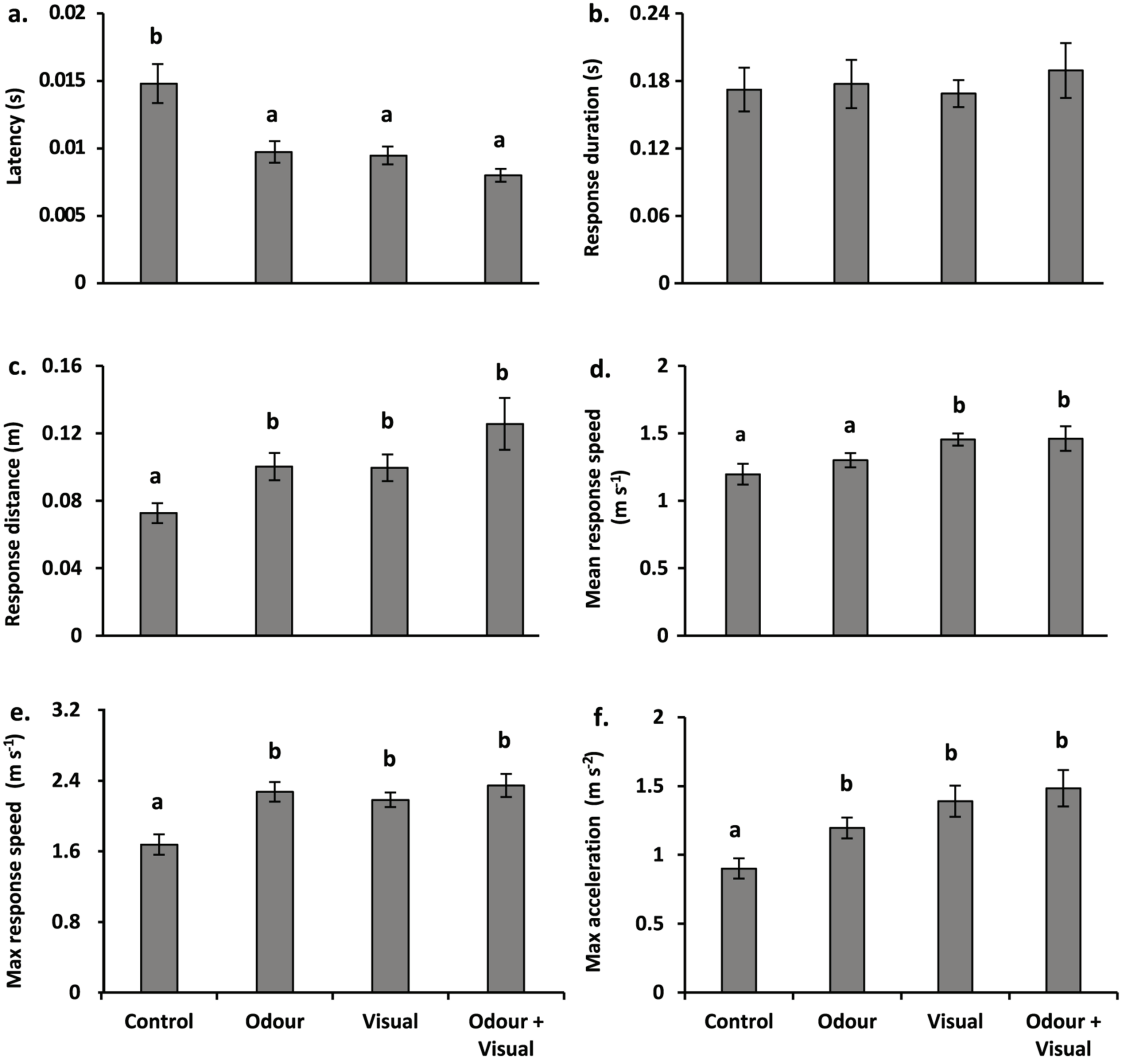
Comparison of fast-start kinematics of juvenile *Acanthochromis polyacanthus* to 3 predator cues and controls. (a) latency (s), (b) response duration (s), (c) response distance (m), (d) mean response speed (m s^-1^), (e) maximum response speed (m s^-1^), and (f) maximum acceleration (m s^-2^) (± S.E.). n = 15.

There was a difference in the overall fast-start behaviour of fish in response to the treatments (MANOVA, Pillai’s Trace: F_18, 159_ = 3.4, p < 0.0001; Fig 1). Overall, *A*. *polyacanthus* juveniles responded to the burst stimulus more effectively when they had been exposed to a predator cue, regardless of whether it was the odour, sight or a combination of the two cues.

Latency to respond differed among the four treatments (ANOVA: F_3, 56_ = 9.35, p < 0.0001; *d* = 1.43), and post-hoc tests indicated that latency was significantly reduced in the presence of any predator cue compared to controls (Fig 1a), with all predator treatments eliciting a similar response from *A*. *polyacanthus*. Response distance, mean response speed, maximum response speed and maximum acceleration showed a similar pattern of response, with significant differences evident between controls and predator treatments (response distance, F_3, 56_ = 6.25, p = 0.0009, *d* = 1.13, Fig 1c; mean response speed, F_3, 56_ = 6.13, p = 0.0011, *d* = 1.53, Fig 1d; maximum response speed, F_3, 56_ = 7.395, p = 0.0003, *d* = 1.36, Fig 1e; maximum acceleration, F_3, 56_ = 6.55, p = 0.0007, *d* = 1.27, Fig 1f). For response duration, maximum response speed and maximum acceleration, responses from control fish were significantly lower than achieved by fish exposed to one of the predator-cue treatments, which did not differ from one another (Tukey’s results in Fig 1c, 1e and 1f). Mean response speed displayed a similar trend, but for this variable fish exposed to predator odour did not show an increase in response above that of the controls (Fig 1d). Response duration was the only variable found to show no significant difference among treatments (F_3, 56_ = 0.001, p = 0.891; 1b), even though the strength of the trend was moderately high (as indicated by its effect size, *d* = 0.71).

## Discussion

The results from this study show that individuals that are forewarned of a predator are able to adjust their level of responsiveness and modify their escape responses. When predator cues were present, kinematic aspects of the prey’s fast-start changed in a way that maximised the probability of escape. For instance, escape latency was greatly reduced while maximum response speed was greatly increased in the presence of predator cues.

Findings from this study mirror that of previous work, which has shown high variability within escape responses [8, 9, 13]. Fast-starts consist of three discrete kinematic stages [25]: the formation of the c-bend (stage 1), the propulsive stroke (stage 2) and a variable stage involving continuous swimming or coasting (stage 3). Our findings suggest that fast-start responses are under more behavioural control than previously thought, are highly variable and context dependent, as suggested by previous studies [11], suggesting that prey are able to adjust their escape responses in relation to the perceived risk. What this means is that if the initial threat is considered to be high risk (e.g., both a visual and olfactory cue presented) then prey modulate their response in relation to this threat (e.g., reduced latency resulting in a rapid locomotory response). This follows “The Economic Hypothesis” whereby prey are suggested to alter their behaviour (to result in the lowest cost to overall fitness) depending on their perceived level of risk [15]. We suggest that the overall fast-start response involves a number of steps in a sequence that are more likely to be under behavioural control. More specifically, while stage 1 (the formation of the c-bend) must be carried through to completion and can be thought of as more hardwired, fast-start responses are modified in stages 2 and 3 allowing for a variable response dependent upon the level of the threat presented.

Our study found high levels of within-individual variability among consecutive bursts, with no sign of a reduction or escalation in responsiveness from the first to last elicited burst. These results are similar to others studies [14] where inter-burst variation can exceeded 40% in various components of the escape response. Within-individual variation may be the result of trade-offs between escape and choosing to stay in the same area [10]. Factors such as risk, the cost of the escape response (i.e. loss of foraging opportunity), group sizes [15] and foraging behaviour (i.e. bolder, more exploratory fish devote more time towards foraging than anti-predator vigilance; [26–27]) have been shown to affect one or several steps within the escape response.

Few studies have examined the underlying causes of within-individual variation in fast-start responses. Our findings suggest that at least some of the variation within individual fish may be associated with their level of motivation at the time of each startle. Webb [28] found prey responded maximally to predator attacks that were followed by chases. Similarly, our study shows that forewarning of risk by the presence of predator cues increased the performance of the fast-start response. Prey use as much information as available to categorise the level of local risk [6, 29]. In the present study, the response of *A*. *polyacanthus* to the visual cues of a predator was no different from olfactory cues, or the combination of cues across most kinematic traits. In fact, all measured traits, with the exception of mean response speed, were statistically similar. This is surprising since olfactory cues and visual cues often play slightly different roles in risk categorisation by prey, with chemical cues warning the prey that a known predator is in the vicinity, while visual cues can provide information on a predator’s intent and motivation [17, 30]. In our instance, the predator was confined within a plastic tank, oriented broadside and therefore away from the potential prey. Smith and Belk [31] found the escape performance of mosquitofish, *Gambusia affinis*, relied upon visual cues of their predator during risky behaviours (e.g. foraging in the presence of a predator). In our experiment the prey may have perceived the dottyback as a disinterested predator due to its orientation and distance from the prey, and this may have led to the lack of threat escalation when visual cues were available.

Our results strongly suggest that prey are responsive to perceived risk and modulate their fast-start responses perhaps to minimise the energy expended. This suggests that optimisation is worthwhile despite the immense cost of a wrong decision due to the high energy cost of fast-start performance. The present study highlights the importance of being responsive to olfactory and visual information during the vulnerable juvenile phase, when fish must rapidly learn to recognise predators and classify motivations [4]. Maintaining behavioural flexibility in response to changing environmental cues is important for animals that undergo complex life histories. In diverse environments such as coral reefs, fishes are constantly exposed to visual cues and chemical odours and learning the relevance of the information encapsulated in these cues is crucial for their survival [4, 20]. The present study highlights the importance for naïve reef fishes of assessing both visual and olfactory cues to alter the amplitude of their kinematic responses in reaction to a posed threat.

## Supporting Information

S1 Dataset All data which is used in this study. (XLSX)
